# When the Antibiogram Offers Nothing Else: Ceftriaxone–Sulbactam–Disodium EDTA With Intravenous Colistin in Two Neonates With Metallo‐β‐Lactamase‐Producing *Acinetobacter baumannii* Meningitis

**DOI:** 10.1002/ccr3.73664

**Published:** 2026-11-05

**Authors:** Naini Puri, Shantanu Shubham, Syed Moiz Ahmed, Richa Joshi, Divya Mishra, Hanshmani Prasad Chaudhary

**Affiliations:** ^1^ Department of Pediatrics Graphic Era Institute of Medical Sciences, Graphic Era Deemed To Be University Dehradun Uttarakhand India; ^2^ Department of Obstetrics and Gynaecology Graphic Era Institute of Medical Sciences, Graphic Era Deemed To Be University Dehradun Uttarakhand India; ^3^ Department of Pediatrics Nepal Medical College and Teaching Hospital Kathmandu Nepal

**Keywords:** *Acinetobacter baumannii*, ceftriaxone–sulbactam–EDTA, colistin, metallo‐β‐lactamase, neonatal meningitis, susceptibility breakpoint

## Abstract

Two neonates with metallo‐β‐lactamase‐producing *Acinetobacter baumannii* meningitis, resistant to every tested alternative, received ceftriaxone–sulbactam–disodium EDTA plus intravenous colistin, which, to our knowledge, represents the first reported neonatal use of this combinations. Cultures were sterilized by day 7; both survived with normal assessments at 3‐months. Concomitant colistin precludes attributing the response to either agent.

## Key Clinical Message

In two neonates with metallo‐β‐lactamase‐producing *Acinetobacter baumannii* meningitis resistant to every tested agent, ceftriaxone–sulbactam–disodium EDTA plus intravenous colistin was followed by sterile cultures. Concomitant colistin precludes attributing the response to either agent. The choice was laboratory‐driven, not evidence‐based, and requires component‐specific monitoring and prolonged follow‐up.

## 1 Introduction

*Acinetobacter baumannii* is a leading cause of late‐onset neonatal sepsis in South Asia. In the Delhi Neonatal Infection Study, multidrug resistance was documented in 82% of *Acinetobacter* isolates [1], and in the multicountry NeoOBS cohort, 71% of *Acinetobacter* blood isolates were carbapenem‐resistant with 28‐day mortality of 17.7% among culture‐positive infants [2]. Meningitis due to this organism is uncommon but disproportionately lethal: 17 of 51 patients died in one series [3]. Once metallo‐β‐lactamase (MBL) production removes carbapenems, the agents current evidence favors—ceftazidime–avibactam with aztreonam, sulbactam–durlobactam, and tigecycline—are frequently untestable in resource‐limited laboratories. Only 4 of 40 antibiotics licensed for adults since 2000 carry neonatal dosing information [4].

CSE‐1034 (Elores) combines ceftriaxone, sulbactam, and disodium EDTA; developed by Venus Remedies Limited (Panchkula, India) and launched in 2013, its Indian marketing rights passed to Cipla Limited (Mumbai, India) in October 2019. Sulbactam inhibits serine β‐lactamases and has intrinsic anti‐*Acinetobacter* activity; EDTA chelates the zinc required for MBL catalysis and disrupts biofilm [5]. Independent authors have questioned whether such fixed combinations were adequately evaluated for inhibitor potency and dose adequacy before marketing [6]. Ceftriaxone is generally avoided in neonates because it displaces bilirubin from albumin [7] and precipitates with intravenous calcium, an interaction associated with seven infant deaths among nine reported events [8].

We report two neonates in whom this combination was used, together with intravenous colistin, because the antibiogram offered nothing else that had been tested.

## 2 Case History and Examination

### 2.1 Case 1

A female infant was delivered by caesarean section at 37 weeks for fetal distress, with a birth weight of 2545 g, Apgar scores of 7 and 9, with no maternal fever, prolonged rupture of membranes, or chorioamnionitis. She was outborn and spent 4 days in another unit's neonatal intensive care unit for transient tachypnoea, receiving intravenous ampicillin and gentamicin for 5 days; a sepsis screen and blood culture there were negative, and she was discharged on day 5.

She re‐presented on day 7 with lethargy, temperature instability, a full anterior fontanelle, reduced oral intake for 24 h, and moderate respiratory distress (Downes score 4); tone and movements were normal. The sepsis screen was positive on three of five parameters (C‐reactive protein 34 mg/L, total leucocyte count 4.4 × 10^9^/L, absolute neutrophil count 1.0 × 10^9^/L). Blood and cerebrospinal fluid cultures were both positive. She required non‐invasive ventilation for 3 days and continuous positive airway pressure for two more. No central vascular access was in situ at any point.

### 2.2 Case 2

A male infant was delivered vaginally at 31 weeks, birth weight 1365 g, Apgar scores of 6 and 8, after spontaneous preterm labor with rupture of membranes for 36 h and intrapartum fever (maternal leucocyte count 12.8 × 10^9^/L).

He was stabilized on continuous positive airway pressure (fraction of inspired oxygen ≤ 0.30, no surfactant). A chest radiograph on day 1 suggested pneumonia. A sepsis screen at 6 h was negative; a repeat at 18 h was positive on two parameters (C‐reactive protein 24 mg/L, immature‐to‐total neutrophil ratio above 0.2). On day 2 he developed multiple apnoeas and feed intolerance and was escalated to non‐invasive ventilation; enteral feeds were withheld for 48 h and then graded up. No seizures or tone abnormality were documented, and no central vascular access was in situ. Blood and cerebrospinal fluid cultures obtained on day 2 were both positive.

## 3 Methods

### 3.1 Differential Diagnosis

Concordant blood and cerebrospinal fluid isolates with markedly abnormal cerebrospinal fluid indices argued against contamination in both infants. In Case 1, the negative screen and culture at the referring hospital supported acquisition of a new organism rather than persistence of unrecognized infection.

### 3.2 Investigations

Blood cultures were processed on the automated BacT/ALERT system; cerebrospinal fluid was obtained by lumbar puncture before escalation of therapy in both infants. Identification and susceptibility used the VITEK 2 system (bioMérieux, Marcy‐l'Étoile, France) with the GN identification and AST cards, interpreted per Clinical and Laboratory Standards Institute M100, 33rd edition (2023) [9]. MBL production was confirmed by the imipenem–EDTA combined disk test. Cerebrospinal fluid indices are given in Table 1 and the antibiogram in Table 2. Ceftazidime–avibactam, ceftazidime–avibactam with aztreonam, and tigecycline were not determined (Table 2).

**TABLE 1:** Cerebrospinal fluid and blood culture findings before and after 7 days of therapy.

Parameter	Case 1 baseline	Case 1 day 7	Case 2 baseline	Case 2 day 7	Reference, ≤ 28 days
CSF white cell count (cells/mm^3^)	180 (90% PMN)	20 (lymphocytic)	220 (85% PMN)	15 (lymphocytic)	≤ 15
CSF protein (mg/dL)	180	70	200	65	≤ 127
CSF glucose (mg/dL)	18	50	20	55	≥ 25
Paired blood glucose (mg/dL)	80	—	85	—	—
CSF culture	*A. baumannii*	Sterile	*A. baumannii*	Sterile	—
Blood culture	*A. baumannii*	Sterile	*A. baumannii*	Sterile	—

*Note:* Both infants received 21 days of CSE‐1034 with intravenous colistin; sterilization reflects the combination. Blood cultures were drawn on the same day as the day‐7 lumbar puncture. Reference values are age‐specific bounds for infants aged 28 days or less [10].

Abbreviations: *A. baumannii* , *Acinetobacter baumannii* ; CSF, cerebrospinal fluid; PMN, polymorphonuclear leukocytes.

**TABLE 2:** Antimicrobial susceptibility profile of both isolates.

Antimicrobial agent	Case 1	Case 2
Ampicillin–gentamicin	R	R
Piperacillin–tazobactam	R	R
Amikacin	R	R
Cefotaxime/ceftazidime	R	R
Meropenem	R	R
Co‐trimoxazole	R	R
Colistin^a^	I (MIC 2 μg/mL)	I (MIC 2 μg/mL)
Ceftriaxone–sulbactam–disodium EDTA (disc diffusion)^b^	Meets manufacturer criterion (nonstandardized; zone diameter not recorded)	Meets manufacturer criterion (nonstandardized; zone diameter not recorded)
Ceftazidime–avibactam^c^	Not tested	Not tested
Ceftazidime–avibactam with aztreonam (synergy)^c^	Not tested	Not tested
Tigecycline^c^	Not tested	Not tested
ESBL (phenotypic)	Not detected	Not detected
MBL (phenotypic, imipenem–EDTA combined disc)	Positive	Positive

*Note:* Identification and susceptibility testing were performed on the VITEK 2 automated system (bioMérieux, Marcy‐l'Étoile, France) from BacT/ALERT blood culture and from cerebrospinal fluid, interpreted according to CLSI M100, 33rd edition [9].

Abbreviations: CLSI, clinical and laboratory standards institute; EDTA, ethylenediaminetetraacetic acid; ESBL, extended‐spectrum β‐lactamase; EUCAST, european committee on antimicrobial susceptibility testing; I, intermediate; MBL, metallo‐β‐lactamase; MIC, minimum inhibitory concentration; R, resistant.

^a^ Determined by broth microdilution, the only method CLSI recognizes as valid for polymyxins. CLSI assigns Acinetobacter spp. no susceptible category for colistin, classifying MICs of 2 μg/mL or below as intermediate and 4 μg/mL or above as resistant [9].

^b^ No CLSI or EUCAST interpretive breakpoint exists for the fixed ceftriaxone–sulbactam–disodium EDTA combination. Testing used disc diffusion with a manufacturer‐supplied disc read against the manufacturer's interpretive criterion; the laboratory recorded only the categorical interpretation and the measured zone diameters are not available. The result is nonstandardized and not breakpoint‐validated, and is deliberately not reported as “S” [11].

^c^ Not tested: the laboratory held no discs, gradient strips, VITEK 2 cards, or synergy‐testing capability for these agents at any point during the study period, and none was on hospital formulary.

VITEK 2 carries no card for the fixed combination, so CSE‐1034 was tested separately by disk diffusion with a manufacturer‐supplied disk, read against the manufacturer's zone criterion. The laboratory reported only the categorical interpretation; the measured zone diameters were not recorded and could not be retrieved retrospectively, which we record as a limitation. Neither CLSI nor EUCAST publishes a breakpoint for this combination, and laboratories reporting it commonly apply ceftriaxone zone diameters as a surrogate [11]. The result is therefore recorded in Table 2 as “meets manufacturer criterion (nonstandardized; zone diameter not recorded)”, not as a breakpoint‐validated category, and should not be read as equivalent to CLSI “susceptible”. Colistin was tested by broth microdilution, the only method CLSI accepts for polymyxins [9]; the minimum inhibitory concentration was 2 μg/mL in both isolates, reported as intermediate.

### 3.3 Treatment

Both infants received intravenous CSE‐1034 60 mg/kg of the ceftriaxone component every 12 h with intravenous colistimethate 2.5 mg/kg (colistin base activity) every 12 h for 21 days, each infused over 30 min, without a loading dose and without intrathecal or intraventricular colistin. Calcium‐containing infusions were paused around each ceftriaxone dose with calcium‐free flushes before and after; the two agents were never given simultaneously through the same line [8].

Before treatment, parents were specifically counseled that CSE‐1034 is not licensed for neonates, that no neonatal safety or efficacy data exist, and that its use was exceptional; written consent to off‐label treatment was obtained in addition to consent for publication [12]. The decision was taken jointly by the treating neonatologists and the clinical microbiologist and was reviewed by the hospital antimicrobial stewardship committee.

#### 3.3.1 Dose Derivation and Its Limitations

Elores is supplied as a 1.5 g vial containing 1000 mg ceftriaxone, 500 mg sulbactam, and 37 mg disodium EDTA in a fixed 2:1 ratio [13]. The regimen delivered 120 mg/kg/day ceftriaxone, 60 mg/kg/day sulbactam, and approximately 4.4 mg/kg/day disodium EDTA.

The dose is that published for pediatric practice: in the post‐marketing surveillance study of 2500 patients, 750 were pediatric, and the pediatric dose is reported as 30–75 mg/kg, rising to 120 mg/kg in severe infections [14], the upper limit we applied for culture‐proven meningitis with bacteremia. A drug‐use analysis of 450 children with hospital‐acquired pneumonia recorded 17 adverse events in its infant group, all local or mild [15].

Three limitations must be stated plainly. First, no neonatal pharmacokinetic or pharmacodynamic data exist for this combination: the surveillance cohort had a mean age of 11.85 ± 3.28 years [14], and the pneumonia analysis did not separately report neonates [15]. Neonatal data exist only for the components. Ceftriaxone given to neonates at 50 mg/kg once daily has an elimination half‐life of about 15 h, longest in the first days of life and in preterm infants [16], so 120 mg/kg/day in two divided doses, imposed by the fixed ratio, exceeds any neonatal‐studied regimen and risks accumulation. Second, the fixed ratio prevents independent optimization of sulbactam. Pooled pediatric population modeling suggests 100–200 mg/kg/day of sulbactam, preferably by extended infusion, for *A. baumannii* [17]; a cerebrospinal‐fluid model indicates approximately 200 mg/kg/day in infants with meningeal inflammation to reach 90% probability of target attainment [18]; and consensus pediatric dosing for carbapenem‐resistant *A. baumannii* is similar [19]. This product delivered 60 mg/kg/day; even 100 mg/kg/day would require 200 mg/kg/day of ceftriaxone, indefensible in a neonate. Adequate sulbactam exposure was therefore probably not achieved—the practical expression of the dose‐adequacy concern raised about fixed unconventional combinations [6]. Third, Indian product literature advises against use in premature infants. Case 2, born at 31 weeks with immature clearance and lower albumin binding capacity, faced greater risk of ceftriaxone accumulation and bilirubin displacement, and was treated outside that recommendation, knowingly, because nothing else testable was active.

#### 3.3.2 Component‐Specific Hazards and the Monitoring They Dictate

CSE‐1034 is three agents, each with its own toxicity, and no neonatal safety study exists; monitoring was constructed component by component (Table 3).

Ceftriaxone contributes bilirubin displacement [7], calcium precipitation [8], biliary sludging in approximately 17% of treated children [20], nephrolithiasis in 1.4% of 284 children [21], and—the hazard most likely to be fatal and least likely to be recognized—immune hemolytic anemia, with 64% mortality in children [22]. Neither infant had prior ceftriaxone exposure, which reduces but does not eliminate that risk. Urine hemoglobin and direct antiglobulin testing were not performed; the component hazard with the highest pediatric mortality was therefore not actively screened for, and a stable hemoglobin cannot exclude subclinical hemolysis.

Sulbactam contributes β‐lactamase inhibition and intrinsic anti‐*Acinetobacter* activity, penetrates inflamed meninges [23], and is the least problematic component in a neonate.

Disodium EDTA has the least neonatal reassurance. It chelates the zinc required for MBL catalysis and disrupts biofilm [5], and that same property is its hazard: disodium EDTA (Na_2_EDTA), unlike calcium disodium EDTA, binds serum calcium. The Centers for Disease Control and Prevention reported three deaths from chelation‐related hypocalcemic cardiac arrest and advised that Na_2_EDTA not be given to children [24], and a five‐year‐old died after receiving the disodium rather than the calcium disodium salt [25]. Chelation delivers gram quantities, whereas this product delivers approximately 4.4 mg/kg/day, and no hypocalcaemic event has been reported in any CSE‐1034 series; whether the adjuvant dose carries any calcium‐binding risk in a neonate with limited parathyroid reserve is unknown, and two monitored infants cannot answer it. Serum calcium was monitored, and because chelation‐induced hypocalcaemia manifests electrically before clinical signs, weekly electrocardiography with corrected QT interval measurement was performed throughout.

**TABLE 3:** Component‐specific hazards of the administered regimen and the monitoring each dictates.

Component	Contribution to activity	Principal hazard in a neonate	Monitoring	Observation in these two cases
Ceftriaxone	Bactericidal PBP inhibition; reliable CSF penetration with meningeal inflammation	Bilirubin displacement from albumin [7]	Serial total and conjugated bilirubin	Reversible conjugated rise, peak day 21
		Precipitation with calcium; fatal cardiopulmonary events in infants [8]	Line separation; never administered simultaneously	Protocol applied; no event
		Biliary sludging in approximately 17% of treated children [20]	Conjugated bilirubin; abdominal ultrasonography	Ultrasonography normal at end of treatment in both
		Nephrolithiasis in 1.4% of 284 children treated at 75 mg/kg [21]	Ultrasonography of kidneys, ureters and bladder	Normal in both at end of treatment
		Immune hemolytic anemia; 64% mortality in children [22]	Urine for hemoglobinuria; LDH; direct antiglobulin test	Not performed (monitoring gap)
Sulbactam	β‐lactamase inhibition; intrinsic anti‐*Acinetobacter* activity; documented CSF penetration [23]	Hypersensitivity; transient transaminase elevation; sub‐target exposure at the fixed ratio [17, 18]	Aminotransferases; no exposure monitoring possible	Aminotransferases normal throughout; exposure not measured
Disodium EDTA	Chelates Zn^2+^ required for MBL catalysis; disrupts biofilm; increases outer‐membrane permeability [5]	Calcium chelation. Na_2_EDTA has caused fatal hypocalcemic cardiac arrest in children at chelation doses and is excluded from pediatric chelation use [24, 25]; neonatal relevance at adjuvant doses unknown	Serum calcium; weekly ECG with QTc	Calcium normal; QTc normal at every weekly assessment
Colistin^a^	Polymyxin activity against carbapenem‐resistant Gram‐negative organisms	Nephrotoxicity; hypomagnesemia; hypokalemia [26]	Creatinine, magnesium, potassium	Creatinine fell; magnesium nadir 0.48 and 0.53 mmol/L, recovered
Meningitis itself	Not applicable	Postinfectious hydrocephalus; extra‐axial collection; late cognitive, motor, hearing and seizure sequelae [27, 28]	Weekly cranial ultrasonography; serial head circumference; audiometry; MRI; structured neurodevelopmental follow‐up	Normal weekly imaging and audiometry; three‐month assessment normal (early follow‐up only)

*Note:* Serial laboratory values supporting this table are reported in Section 4.2. Normal observations in two infants do not establish safety.

Abbreviations: CSE‐1034, ceftriaxone–sulbactam–disodium EDTA; CSF, cerebrospinal fluid; ECG, electrocardiography; EDTA, ethylenediaminetetraacetic acid; LDH, lactate dehydrogenase; MBL, metallo‐β‐lactamase; MRI, magnetic resonance imaging; Na_2_EDTA, disodium ethylenediaminetetraacetic acid; PBP, penicillin‐binding protein; QTc, corrected QT interval.

^a^ Co‐administered agent, not a component of CSE‐1034.

#### 3.3.3 Rationale for Use in Meningitis

CSE‐1034 has been used in bacterial meningitis: of 2500 patients in the surveillance study, 55 had meningitis, including 37 pediatric patients [14]. Ceftriaxone crosses inflamed meninges reliably. Sulbactam has been detected in cerebrospinal fluid during treatment of carbapenem‐resistant *A. baumannii* meningitis [23], although at the exposure delivered here, cerebrospinal‐fluid target attainment is improbable [18]. Disodium EDTA penetration is unknown; if it does not reach the cerebrospinal fluid, the mechanistic rationale is confined to the systemic compartment. Intravenous colistin achieves poor and variable cerebrospinal fluid concentrations, and adjunctive intrathecal colistin has been proposed as more reliable [3]; our infants received intravenous colistin only, which makes the sterilization harder rather than easier to attribute to either agent.

### 3.4 Literature Search for Prior Neonatal Use

PubMed was searched on 20 September 2026 without date or language limits using: (ceftriaxone AND sulbactam AND (EDTA OR edetate)) OR “CSE‐1034” OR “CSE 1034” OR Elores OR “antibiotic adjuvant entity”, returning 19 records; reference lists of every CSE‐1034 publication were hand‐searched. Ten records reported clinical use (Table 4); none described a neonate, the youngest population being the infant subgroup of a pediatric pneumonia analysis in which neonates were not separately identified [15]. Adding neonate, newborn, infant, premature, or preterm returned nothing. The gray literature and non‐indexed journals were not searched, so absolute novelty cannot be established.

**TABLE 4:** Published clinical evidence for CSE‐1034 by patient age.

Study	Design	*n*	Age range	Indication	Affiliation
Mir 2019 (PLEA) [13]	Double‐blind randomized trial	230	Adults	Complicated urinary tract infection	Manufacturer
Chaudhary 2018 [29]	Open‐label phase 3 trial	93	Adults	Community‐acquired pneumonia	Manufacturer
Chaudhary 2017 [14]	Post‐marketing surveillance, 17 centres	2500	All ages; 750 pediatric (mean 11.85 ± 3.28 y)	MDR infections, including 55 with bacterial meningitis (37 pediatric)	Manufacturer
Chaudhary 2018 [15]	Pediatric drug‐use analysis, 17 centres	450	Pediatric only: infants, children and adolescents; neonates not separately reported	Hospital‐acquired pneumonia	Manufacturer
Sathe 2017 [30]	Prospective observational	30	Adults	Ventilator‐associated pneumonia	Independent
Patil 2015 [31]	Retrospective observational	18	12–65 y (mean 48.5 ± 12.1)	MDR septicemia	Independent
Gupta 2016 [32]	Case report	1	20 y	Carbapenem‐ and polymyxin‐resistant sepsis	Independent
Present report	Case series	2	Neonates, days 2 and 7 of life	*A. baumannii* meningitis (with intravenous colistin)	Independent

*Note:* The weight‐based dose derives solely from Chaudhary 2017: 30–75 mg/kg of body weight, rising to 120 mg/kg in severe infections [14]. No neonatal patient was identified in the PubMed search described in Section 3.4; absolute novelty cannot be established. Studies designated “Manufacturer” were authored wholly or predominantly from Venus Remedies Limited or Venus Medicine Research Centre, which developed the product; Indian marketing rights were acquired by Cipla Limited in October 2019.

Abbreviations: CSE‐1034, ceftriaxone–sulbactam–disodium EDTA; MDR, multidrug‐resistant; PLEA, the trial acronym used by the authors of the cited trial; y, years.

## 4 Conclusion and Results

### 4.1 Microbiological Response

Repeat blood cultures and repeat lumbar puncture on day 7 were sterile in both infants, with a falling cell count, a shift from polymorph to lymphocyte predominance, falling protein, and rising glucose (Table 1). Because intravenous colistin was co‐administered throughout, this response is that of the combination and cannot be attributed to CSE‐1034.

### 4.2 Laboratory and Monitoring Course

Conjugated bilirubin rose to a peak of 41.7 μmol/L (2.44 mg/dL) on day 21 in Case 1 and 43.3 μmol/L (2.53 mg/dL) in Case 2, returning to baseline by day 35, while total bilirubin declined steadily in both. Aminotransferases and alkaline phosphatase remained within neonatal limits; gamma‐glutamyl transferase rose modestly and fell, and requires interpretation against neonatal intensive care reference data rather than adult intervals [33]. Serial values are in Table 5.

**TABLE 5:** Serial laboratory values in SI units, with conventional units in parentheses.

Parameter	Case	Baseline	Day 7	Day 14	Day 21	Day 28	Day 35
Conjugated bilirubin, μmol/L (mg/dL)	1	19.3 (1.13)	22.9 (1.34)	31.5 (1.84)	41.7 (2.44)	26.8 (1.57)	20.2 (1.18)
	2	20.0 (1.17)	21.7 (1.27)	32.0 (1.87)	43.3 (2.53)	26.8 (1.57)	20.5 (1.20)
Total bilirubin, μmol/L (mg/dL)	1	145 (8.5)	159 (9.3)	128 (7.5)	99 (5.8)	74 (4.3)	55 (3.2)
	2	162 (9.5)	183 (10.7)	147 (8.6)	115 (6.7)	86 (5.0)	65 (3.8)
AST, U/L	1	36	40	38	34	31	29
	2	48	51	47	43	39	36
ALT, U/L	1	23	27	25	22	20	18
	2	37	41	38	34	30	27
ALP, U/L	1	430	446	424	401	378	356
	2	472	489	465	440	415	392
GGT, U/L	1	255	—	279	290	248	222
	2	263	—	291	259	232	217
Urea, mmol/L (mg/dL)^a^	1	—	3.80 (22.8)	4.68 (28.1)	4.28 (25.7)	3.35 (20.1)	2.73 (16.4)
	2	—	3.18 (19.1)	3.96 (23.8)	3.41 (20.5)	2.70 (16.2)	2.25 (13.5)
Creatinine, μmol/L (mg/dL)	1	—	82 (0.93)	74 (0.84)	60 (0.68)	52 (0.59)	46 (0.52)
	2	—	74 (0.84)	66 (0.75)	57 (0.64)	51 (0.58)	44 (0.50)
Sodium, mmol/L	1	—	135	134	137	136	138
	2	—	138	140	137	139	141
Potassium, mmol/L	1	—	5.7	5.1	4.8	4.6	4.9
	2	—	5.3	5.5	5.0	4.7	5.1
Calcium, mmol/L (mg/dL)	1	—	2.18 (8.7)	2.23 (8.9)	2.28 (9.1)	2.25 (9.0)	2.33 (9.3)
	2	—	2.25 (9.0)	2.33 (9.3)	2.30 (9.2)	2.38 (9.5)	2.35 (9.4)
Magnesium, mmol/L (mg/dL)	1	—	0.58 (1.41)	0.51 (1.25)	0.48 (1.17)	0.50 (1.22)	0.56 (1.35)
	2	—	0.63 (1.52)	0.57 (1.39)	0.53 (1.28)	0.56 (1.36)	0.62 (1.51)
Platelet count, × 10^9^/L	1	—	130	150	155	165	180
	2	—	240	250	260	280	300
ANC, × 10^9^/L	1	—	1.0	1.5	2.3	3.4	4.0
	2	—	6.0	5.2	5.0	4.8	4.5

*Note:* Conversion factors used: bilirubin ×17.1; urea × 0.1665; creatinine × 88.4; calcium × 0.25; magnesium × 0.4114. Gamma‐glutamyl transferase was not measured on day 7. The cholestasis threshold is a conjugated bilirubin above 17 μmol/L (1.0 mg/dL) [34]. Gamma‐glutamyl transferase should be interpreted against neonatal intensive care reference data, in which mean concentrations in neonates without cholestasis, sepsis or liver disease were approximately 140 ± 88 U/L, rather than against adult intervals [33]. Direct antiglobulin testing, urine hemoglobin, and lactate dehydrogenase were not measured; normal values in this table are observations in two infants and do not establish safety.

Abbreviations: ALP, alkaline phosphatase; ALT, alanine aminotransferase; ANC, absolute neutrophil count; AST, aspartate aminotransferase; GGT, gamma‐glutamyl transferase; SI, système international.

^a^ Values are blood urea, not blood urea nitrogen; the conversion factor applied is × 0.1665.

Urea and creatinine followed the expected postnatal decline, and neither infant met criteria for acute kidney injury. Sodium, potassium, and calcium remained normal; magnesium fell to a nadir of 0.48 and 0.53 mmol/L, consistent with hypomagnesaemia described during colistin therapy in preterm infants [26], and recovered without supplementation. Case 1's presenting neutropenia recovered by day 35; platelets remained above 130 × 10^9^/L. Case 2 maintained normal counts.

Weekly electrocardiography showed a normal corrected QT interval at every assessment in both infants. End‐of‐treatment abdominal and renal ultrasonography were normal in both, with no biliary sludge, gallbladder pseudolithiasis, or nephrolithiasis. These are observations in two infants with incomplete hemolysis surveillance; they do not constitute evidence of neonatal safety.

### 4.3 Outcome and Early Follow‐Up

Both infants completed 21 days without discontinuation for any adverse event. Weekly cranial ultrasonography with serial head circumference measurement was normal throughout, with no ventriculomegaly, extra‐axial collection, or abnormal head growth. Brainstem evoked response audiometry before discharge was normal in both. The Hammersmith Neonatal Neurological Examination scored 31 in Case 1 and 29 in Case 2, both optimal; retinopathy screening in Case 2 was normal. No seizures occurred, clinically or on amplitude‐integrated electroencephalography. Magnetic resonance imaging of the brain, before discharge in Case 1 and at 40 weeks' post‐menstrual age in Case 2, was normal. Neurodevelopmental assessment at 3 months was normal in both. This is early follow‐up only; it cannot exclude the sequelae that emerge over years after neonatal meningitis, and both infants remain under structured surveillance.

## 5 Discussion

To our knowledge, and on the basis of the search in Section 3.4, these are the first reported neonatal uses of this combination and the first in neonatal Gram‐negative meningitis. Pediatric use is established, with a published weight‐based dose and documented infant exposure (Table 4) [14, 15], use in the newborn has not been reported, though unindexed reports cannot be excluded.

Case 1 illustrates the commonest route of acquisition: 4 days in another unit, discharge well on day 5, and re‐presentation on day 7 with culture‐proven meningitis, most probably acquired horizontally during the first admission. Case 2 met the definition of early‐onset sepsis, yet *A. baumannii* is characteristically nosocomial; ascending infection is most plausible, but early horizontal acquisition cannot be excluded without typing.

### 5.1 Availability Bias Determined the Choice of Agent

Ceftazidime–avibactam with aztreonam is the established approach to MBL producers, and sulbactam–durlobactam was non‐inferior to colistin for 28‐day mortality in carbapenem‐resistant *Acinetobacter* infection with substantially less nephrotoxicity [35]. Neither was tested, nor was tigecycline [36], because the laboratory held no reagents and none was on formulary—while CSE‐1034 discs were stocked.

We name this an availability bias rather than a therapeutic judgment: the agent was chosen because it was the one for which a result could be generated. Prescribing follows testability silently; no clinician records that ceftazidime–avibactam was not considered because it could not be tested, yet that was the operative reason in both cases. A neonatal unit's prescribing is bounded by its laboratory's panel, and expanding what a laboratory can measure costs a fraction of a new antimicrobial.

### 5.2 Why Attribution Remains Uncertain

Both infants received intravenous colistin throughout, so sterilization cannot be attributed to CSE‐1034; colistin alone, CSE‐1034 alone, their combination, or host response are all compatible with the observed course. The susceptibility result came from a nonstandardized disc without a validated breakpoint, and MBL‐producer susceptibility was only 39.4% in independent testing [11]. The fixed ratio delivered sulbactam well below the exposure pediatric modeling suggests the organism requires [17, 18]. EDTA penetration into cerebrospinal fluid is unknown, and no drug concentrations were measured. In two infants, a favorable spontaneous course cannot be excluded.

### 5.3 Reading the Toxicity of a Fixed Combination

A three‐component product cannot be assessed as though it were one drug. Two component hazards—ceftriaxone‐associated immune hemolysis [22] and Na_2_EDTA‐associated hypocalcemic arrest [24]˒ [25]—are low‐frequency, high‐consequence events that two patients cannot exclude, and the first was not screened for. Chelation and adjuvant exposures differ by orders of magnitude, and whether the disodium salt at this dose has any neonatal relevance is unresolved. Normal calcium and weekly QTc across 21 days in two infants ido not constitute a safety dataset and should not be read as one; its value is to establish what any subsequent report should measure.

The conjugated bilirubin trajectory—rising through weeks two and three, resolving by day 35, with normal aminotransferases—is the pattern of cholestasis and is temporally consistent with ceftriaxone‐associated biliary sludging [20], but sepsis, prematurity, and parenteral nutrition are strong alternative explanations, and end‐of‐treatment ultrasonography was normal. It should not be called proven drug‐induced cholestasis.

### 5.4 Limitations

Two patients, no comparator, colistin in both. Susceptibility rested on a nonstandardized manufacturer criterion without a validated breakpoint, and the measured zone diameters were not recorded; colistin was intermediate rather than susceptible; the agents current evidence would favor were never tested. No pharmacokinetic measurements were made, and sulbactam exposure was almost certainly below pediatric targets. Urine hemoglobin and direct antiglobulin testing were not performed. Neurodevelopmental follow‐up extends only to 3 months, whereas after neonatal meningitis 10.8% of survivors had severe and 9% moderate impairment at 9–10 years [27], 30% had neurodevelopmental impairment in a recent cohort [28], and hearing loss, hydrocephalus, and seizures emerge late; surveillance to at least school age is required before any statement about outcome can be made. The published evidence base for this agent is predominantly manufacturer‐generated (Table 4).

## 6 Conclusion

In two neonates with MBL‐producing *A. baumannii* meningitis resistant to every agent the laboratory could test, ceftriaxone–sulbactam–disodium EDTA with intravenous colistin was followed by sterile cultures by day 7, completion of 21 days, survival, and normal early assessments at 3 months. Because colistin was given throughout, the response cannot be attributed to CSE‐1034, and normal component‐specific monitoring in two infants is not evidence of neonatal safety.

These observations are hypothesis‐generating; they establish neither efficacy nor safety, and the fixed ratio means neither component can be dosed to its own optimum. The regimen should not be used routinely. Its central lesson concerns diagnostic and therapeutic uncertainty: where no tested alternative exists, prescribing is driven by what the laboratory can measure rather than by comparative evidence, and exceptional use then requires explicit off‐label consent, microbiology and stewardship involvement, separation from calcium‐containing infusions, the component‐specific monitoring in Table 3, and neurodevelopmental surveillance well beyond infancy. What a unit can test determines what a unit prescribes.

## Author Contributions

**Naini Puri:** conceptualization, methodology, data curation, investigation, writing – original draft. **Shantanu Shubham:** conceptualization, methodology, supervision, formal analysis, investigation, writing – original draft, writing – review and editing, software. **Syed Moiz Ahmed:** conceptualization, methodology, data curation, supervision, writing – original draft, writing – review and editing, visualization. **Richa Joshi:** conceptualization, methodology, writing – review and editing, writing – original draft, software. **Divya Mishra:** methodology, writing – original draft, writing – review and editing, formal analysis, data curation, investigation. **Hanshmani Prasad Chaudhary:** conceptualization, investigation, writing – original draft, writing – review and editing, methodology, validation, formal analysis, project administration, data curation, resources.

## Funding

The authors have nothing to report.

## Ethics Statement

This is a retrospective case series. Institutional review board approval was waived, as approval is not required at our institution for a retrospective report of care already delivered. The work was conducted in accordance with the Declaration of Helsinki, and written informed consent for publication was obtained as described below.

## Consent

Written informed consent for publication of these cases and the accompanying clinical data was obtained from a parent of each infant, using the *Clinical Case Reports* patient consent form. As both patients are minors, consent was provided by a parent, whose name and relationship to the patient are recorded on each form. A separate signed form was obtained for each of the two patients. Signed originals are retained by the corresponding author and will be provided to the publisher on request. Separately, before treatment, parents of both infants were informed that ceftriaxone–sulbactam–disodium EDTA is not licensed for neonates and that its use was off‐label and exceptional, and gave written consent to treatment.

## Conflicts of Interest

The authors declare no conflicts of interest. Specifically, no author has received honoraria, consultancy fees, speaking fees, travel support, research funding, product supply or support of any other kind from Cipla Limited, which acquired Indian marketing rights for Elores in October 2019, from Venus Remedies Limited or Venus Medicine Research Centre, which developed the product, or from any other manufacturer or distributor of ceftriaxone–sulbactam–disodium EDTA, within the preceding 36 months or at any other time. No author holds any financial interest in these or related entities.

## Data Availability

The data that support the findings of this study are available from the corresponding author upon reasonable request.
